# Reliability and validity of the German version of the Iowa infant feeding attitude scale (IIFAS-G) and relations to breastfeeding duration and feeding method

**DOI:** 10.1186/s13006-024-00665-6

**Published:** 2024-08-21

**Authors:** Debora Suppiger, Giancarlo Natalucci, Tilman Reinelt

**Affiliations:** Family Larsson-Rosenquist Foundation Center for Neurodevelopment, Growth, and Nutrition of the Newborn, Department of Neonatology, University Hospital Zurich and University of Zurich, Frauenklinikstrasse 10, Zurich, 8091 Switzerland

## Abstract

**Background:**

Public health initiatives (e.g., the Baby Friendly Hospital Initiative) have led to an increase in breastfeeding rates worldwide. However, as (exclusive) breastfeeding duration is still below WHO recommendations, it is crucial to understand the factors that influence decisions on breastfeeding practice. Modifiable psychological factors such as intention to breastfeed have therefore become targets of recent interventions. As the intention to breastfeed is among the strongest predictors of breastfeeding duration, reliable tools for measuring the intention to breastfeed are needed. The Iowa Infant Feeding Attitude Scale (IIFAS) measures attitudes towards infant feeding and is used in various languages and across different cultural contexts. However, there has been no German version of the IIFAS (IIFAS-G) so far. The aim of this study was to investigate reliability, validity, and associations of the IIFAS-G with feeding method and breastfeeding duration.

**Methods:**

Between August and November 2022, a total of 353 mothers (*M*_age_ = 35 years, *SD*_age_ = 4.2 years) of singleton infants (47.3% female (1 undetermined), *M*_age_ = 10.8 months, *SD*_age_ = 4.7 months, age range: 3-547 days; 90.4% living in Switzerland) participated in an online survey. The IIFAS-G was administered as a part of a larger study on early child development and infant feeding method.

**Results:**

The translated IIFAS-G showed unsatisfactory model fit for the two factor 17-item solution. Four items showed low factor loadings. After item reduction, a 13-item two factor solution showed satisfactory model fit (CFI = 0.92, TLI = 0.90, RMSEA = 0.07) and high internal consistency (Cronbach’s α = 0.85). The IIFAS-G score was higher for mothers who exclusively breastfed their infants compared to mothers who additionally or exclusively fed infant formula. Moreover, mothers with higher IIFAS-G scores were less likely to stop breastfeeding their child over the course of 1.5years (HR = 0.87).

**Conclusion:**

A shorter two-factor IIFAS-G is proposed to investigate attitudes towards breastfeeding and formula feeding in German-speaking mothers.

**Supplementary Information:**

The online version contains supplementary material available at 10.1186/s13006-024-00665-6.

## Background

Breastfeeding is widely considered the ideal way to provide infants with the necessary nutrients for growth and development [1]. The World Health Organization (WHO) recommends six months of exclusive breastfeeding and continued breastfeeding alongside complementary food up to two years of life [2]. Despite the WHO recommendations and many health-related advantages, breastfeeding rates are sinking worldwide [3]. This is especially the case in Europe, where the WHO reports some of the lowest exclusive breastfeeding rates [4]. In German-speaking countries, the rate of exclusive breastfeeding up to six months of age is low, varying from 26% in Switzerland [5], between 8.3% and 16% in Germany [6, 7], and 9.7% in Austria [6]. Even though there have been interventions by the public health system to promote breastfeeding (e.g., Baby Friendly Hospital Initiative (BFHI) or Becoming Breastfeeding Friendly (BBF)), which lead to an increase in initial breastfeeding rates [5, 6, 8, 9], overall breastfeeding rates and duration are still below the recommendations of the WHO [10].

There are numerous reasons why mothers decide to stop breastfeeding their child, encompassing a spectrum of factors from insufficient milk supply [11–13], latching problems of the infant, nipple pain [12, 13], or delivery method [11], to maternal health problems such as obesity [14, 15] or depression [16]. Moreover, social factors like the availability of support [14, 17], returning to work [11, 12], or having the perception that their infant is not satisfied with human milk alone [18] contribute to the decision of breastfeeding cessation. Furthermore, breastfeeding duration is associated with a set of demographic variables including maternal age and education [11]. Recent studies highlighted the importance of psychological factors that include breastfeeding education [17, 19, 20], breastfeeding intention, breastfeeding self-efficacy [14, 21, 22] and attitudes towards breastfeeding [8, 19, 23]. In accordance with the Theory of Planned Behaviour [24], breastfeeding attitudes describe the beliefs an individual holds about breastfeeding and their respective value (e.g., the extent to which one believes that breastfeeding is healthier for babies than infant formula feeding). Breastfeeding attitudes – together with the experience of social norms about breastfeeding and the perceived degree of control over whether or not to breastfeed – are directly related to breastfeeding intention [25], which subsequently influences actual breastfeeding behaviour [23]. Interventions targeting to improve breastfeeding education, modify intention, attitudes, or self-efficacy have been associated with increased (exclusive) breastfeeding rates at birth and the first few months of life [17, 20, 26]. To ensure a sufficient evaluation of such interventions, validated instruments measuring these underlying factors – and change in these factors due to the intervention – are needed.

### Measuring breastfeeding attitudes

The Iowa Infant Feeding Attitude Scale (IIFAS) is a reliable and valid questionnaire that measures attitudes towards breastfeeding. It was developed by de la Mora and colleagues in 1999 in response to declining breastfeeding rates in the United States with the goal to develop an instrument that is easily administered among all types of educational backgrounds. The scale has since been translated into many different languages and used both pre- and postnatal on mothers and fathers among different cultural backgrounds to measure effects of attitudes on feeding method (exclusive, partial breastfeeding vs. infant formula feeding) and breastfeeding duration [e.g., 27–30]. However, there is currently no validated version available for German-speaking countries.

### The present study

The aim of this study was to translate the IIFAS into German (IIFAS-G) and validate the scale in a sample of German-speaking mothers. We hypothesized that the IIFAS-G shows a similar factor structure and reliability as previous translations. Thereby, the IIFAS-G demonstrates to be a reliable and valid instrument to measure attitudes towards breastfeeding. To further demonstrate criterion validity, we hypothesized that IIFAS-G scores can distinguish between mothers who are breastfeeding compared to formula feeding, and are positively associated with overall breastfeeding duration.

## Methods

### Procedure

This study was part of a larger study on early child nutrition, child development, and parenting behaviour, taking place in Switzerland between August and November 2022, with the goal of testing the validity of a variety of German translations and short versions of questionnaires used in other studies related to nutrition and child development. German-speaking parents of infants up to 1.5 years were eligible for participation, which includes parents either currently breastfeeding or formula-feeding. In a large study on Baby Friendly Hospitals, the average breastfeeding duration in Switzerland was 34.8 weeks [31], and in a nationwide study, the probability to breastfeed longer than 10 months was 25% [5]. It was assumed that at 1.5 years most mothers had stopped breastfeeding. Thus, including infant’s up to 1.5 years of age ensures that the sample includes mothers with longer than average breastfeeding duration, but reduces the risk of a potential recall bias regarding breastfeeding behaviour. Participants were contacted if they had given birth at the University Hospital Zurich, a large level three perinatal centre in Zurich, Switzerland, and had consented to be contacted for study purposes. In addition, parents were approached if they had given permission to be contacted for research projects in the department of Developmental Psychology at the University of Zurich. Third, advertisements were placed on social media. Participants were informed that the study aims were to investigate challenges in parenting, in particular concerning the second year of the COVID-19 pandemic, and to test questionnaires for a larger longitudinal study on early nutrition, parenting and child development. As an incentive, a total of 10 gift vouchers worth 50 CHF each could be won in a raffle. In a first part, the participants filled in a baseline questionnaire on early child nutrition, attitudes towards breastfeeding and formula feeding, parental investment, child regulation and response to the COVID-19 pandemic. The questionnaire lasted approximately 40 min. In a second part, a 10-day evening diary (5–10 min per day) was administered. The current study only relied on data from the baseline questionnaire. All data was acquired post-partum.

## Measures

### The IOWA infant feeding attitude scale

The IIFAS is a self-administered scale to assess attitudes towards breastfeeding [32]. The scale consists of 17 items that are rated on a five-point Likert scale from 1 (strongly disagree) to 5 (strongly agree). Nine items are worded favourable towards formula feeding, the remaining eight items are favourable towards breastfeeding. Items favourable towards formula feeding are reverse-coded. Total scores range from 17 to 85. Lower scores indicate positive attitudes towards formula feeding, whereas higher scores indicate more positive attitudes towards breastfeeding. The IIFAS was translated from the original English version into German and then translated back to English to ensure accuracy by two independent researchers who were native speakers of the target language and fluent in the source language. The translated second English version was then compared to the original one in a consensus meeting on terms of general understanding and verbatim translation.

### Infant feeding method

Method of feeding was assessed via questions that included primary infant feeding method (i.e., breastfeeding or formula feeding), duration of breastfeeding (with a distinction between exclusive breastfeeding and additional breastfeeding) and mode of breastfeeding (i.e., breastfeeding and/or bottle-feeding). The items were adapted from the Swiss Infant Feeding Study (SWIFS) [5] and the Infant Feeding Practices Study II (IFPS2) [33]. For an overview, see Table S1 [Online_Supplement.pdf].

### Data analysis

De la Mora et al. did not investigate the underlying internal structure of the IIFAS in their developing process [32]. As items are either formulated to be favorable towards breastfeeding or formula-feeding, a two-factor structure is supposed, which was assessed with a confirmatory factor analysis (CFA). Unfit items were defined as having a negative loading or factor loadings below 0.30 as proposed by Nunnally and Bernstein [34]. The same criterion had been used in the Spanish and Hungarian translations of the of the IIFAS [35, 36]. Model fit was evaluated by Comparative Fit Index (CFI), Tucker-Lewis Index (TLI) and Root Mean Square Error of Approximation (RMSEA).

To assess construct validity and reliability of the IIFAS-G we implemented two different strategies: (1) corrected item-total correlations and (2) Cronbach’s alpha. Items that showed item-total correlations below 0.22 were deemed as poorly functioning as they fall below the item-total correlations (0.22–0.68) of the original study [32]. Similarly, an increase in the alpha coefficient of more than 0.10 when an item is dropped, as proposed by Nanishi and Jimba [30] was interpreted that the respective item assesses a different construct and therefore should be dropped to ensure homogeneity of the scale. With regard to the whole translation, a Cronbach’s alpha value of 0.70 or higher would be considered as acceptable reliability [34].

Exclusive breastfeeding was defined as the mother reporting at the time of the questionnaire that their child was currently exclusively receiving human milk and had never received formula since birth. This includes mothers pumping human milk and giving it via bottle. Formula feeding was defined as receiving formula exclusively or additionally to human milk at any point in time. To assess criterion validity, the mean IIFAS-G scores of both groups were compared using Welch’s two sample t-test.

Lastly, a multivariate Cox-regression was estimated to assess whether the IIFAS-G score was associated with breastfeeding duration. The Cox-regression estimates the probability of breastfeeding cessation for different attitudes towards breastfeeding (IIFAS-G) over time. Since there are several factors known to be associated with breastfeeding duration, potential confounders were entered into the model. These included maternal age, child age, gestational age at birth, maternal education, and current working status.

Due to the high number of missing values on the maternal age covariate, sensitivity analyses without maternal age as a covariate were performed. All analyses were conducted with R Studio Version 4.1.3.

## Results

### Sample characteristics

A total of 536 parents answered the questionnaire. Participants who did not answer at least half of the questions about the IIFAS-G were removed (*n =* 122), as well as participants with children older than 1.5 years (*n =* 44). Fathers were excluded from the sample due to low participation numbers (*n* = 17). The final sample consisted of *N =* 353 mothers. Main residence was Switzerland (*n =* 319; 90.4%). In total, 44.48% of the mothers had a migration background, i.e., were born outside the country of residence. More than half (63.71%) of the mothers were primiparous. The sample consisted of highly educated individuals of which 72.52% hold a degree of tertiary education (of which 36.26% hold a Master’s and 17% a doctoral degree as their highest degree). Furthermore, most mothers were working either full (13.31%) or part-time (56.94%) and had a higher than average household income than families in Switzerland with children below 4 years (approximately 8300 CHF) [37]. Among the participants’ children, 47.31% were female (*n =* 167) and their average age was *M*_*age*_ = 10.84 months, (*SD*_*age*_*=* 4.66 months).

See Table 1 for a detailed sample description.

**Table 1:** Sample characteristics

	M	SD
mother age^a^ (years)	34.99 range = 22–51	4.21
primipara	34.68	4.28
multipara	35.53	4.05
child age (months)	10.84 range = 0–18	4.66
gestational age at birth (weeks)	39.38 range = 24–44	2.14
	** N**	**%**
parity		
primipara	223	63.17
multipara	130	36.83
residence		
Switzerland	319	90.37
other	34	9.63
migration background^c^		
yes	196	55.52
no	157	44.78
educational level		
≥ tertiary	256	72.52
< tertiary	97	27.48
employment status		
full-time employment^d^	47	13.31
part-time employment	192	54.39
maternity leave	75	21.25
other (e.g. school, infrequent working, unpaid leave)	23	6.52
unemployed	16	4.53
monthly household income after taxes in CHF^e^		
< 3’300	12	3.41
3’300–4’300	9	2.56
4’300–5’300	15	4.26
5’300–6’300	27	7.67
6’400–7’500	38	10.80
7’500–8’700	32	9.09
8’700–10’100	51	14.49
10’100–12’000	50	14.20
12’000–15’300	51	14.49
> 15’300	46	13.07
preferred not to answer	13	3.69
child gender		
female	167	47.31
male	185	52.41
not defined	1	0.28

^a^ 30 mothers reported implausible age values (e.g., age = 0 years or 130 years)

^b^ for one child an implausible gestational age at birth was entered (9 weeks)

^c^ migration background was defined as being born outside the country of residence

^d^ at least 90% of the regular working time (e.g., 37.8 h/week in Switzerland)

^e^ income was assessed based on the categories of the European Social Survey; currencies other than CHF were transformed to the equivalent CHF categories

### Factor structure and reliability

**Fig. 1:**
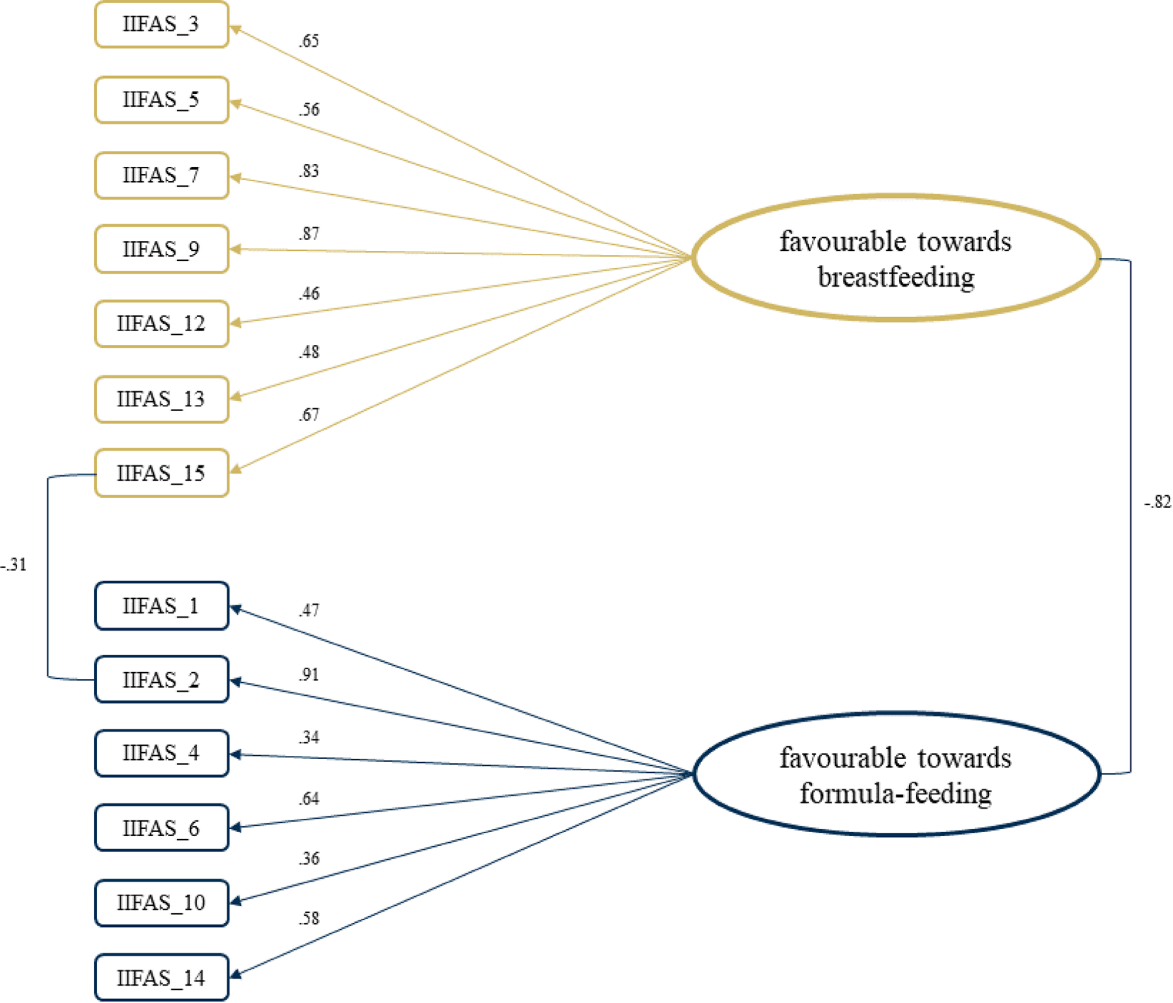
Confirmatory factor analysis of reduced 13-item IIFAS-G. CFA fitting two latent factors (favourable towards breastfeeding vs. favourable towards formula-feeding). Four items (i.e., IIFAS_8, IIFAS_11, IIFAS_16, IIFAS_17) were removed from the model due to low factor loadings

The CFA for a two-factor model with two latent factors (“favorable towards breastfeeding” vs. “favorable towards formula-feeding”) resulted in unsatisfactory model fit (CFI = 0.83; TLI = 0.81; RMSEA = 0.08; BIC = 15529.25); for more details, see Table S3 [Online_Supplement.pdf]. Given the low factor loadings (λ ≤ 0.30) and item-total correlation (< 0.22), items 8, 11, 16, and 17 were eliminated. In addition, an allowance was added for error term covariation in the model for item 2 and 15, as these items covaried most likely due to their similar wording, see Table S2 [Online_Supplement.pdf]. Cronbach’s α was between 0.80 and 0.84 if the item was deleted and thus not considered as an item elimination criteria. The reduced 13-item two-factor solution (see Fig. 1) yielded satisfactory model fit (CFI = 0.92; TLI = 0.90; RMSEA = 0.07; BIC = 11834.74) with high internal consistency (Cronbach’s α = 0.85). Item-Total correlation of all items ranged between 0.41 and 0.77, see Table S4 [Online_Supplement.pdf]. Overall correlation between the two latent factors was *r =* -0.82, which was highly significant (*p* < 0.001). With the reversed-scored 13 items, the overall IIFAS-G score was *M =* 47.22 (*SD =* 8.05) and ranged from 26 to 65.

For an overview of mean and standard deviation, see Table S2 [Online_Supplement.pdf].

### Criterion validity

Of the 350 mothers reporting on their feeding method, 47.42% mothers were currently feeding human milk (*n* = 166), 48.29% had already stopped breastfeeding (*n* = 169), and 4.29% never fed human milk (*n* = 15). Of all the children currently or previously receiving human milk 21.49% were exclusively breastfed (*n* = 72). Of those, most were exclusively fed on the breast (*n* = 97) or predominantly breastfed with some pumped milk given with the bottle (*n* = 62).

Mothers exclusively breastfeeding their child on average had higher IIFAS-G scores than formula-feeding mothers (*M* = 52.45, *SD* = 5.82 vs. *M* = 43.91, *SD* = 7.52), *t*(331.27) = 11.81, *p* < 0.001, *d* = 1.22).

With regard to the recommendations of the WHO, which suggest exclusive breastfeeding for the first 6 months and continued breastfeeding along with complementary foods until 2 years of age, we analysed whether the IIFAS-G is associated with the probability of breastfeeding cessation over the course of 1.5 years. The multivariate Cox-regression revealed the IIFAS-G scores to be highly influential with regard to general breastfeeding duration even when considering confounders such as infant age, gestational age at birth, education or working status. The increase of one point in the IIFAS-G, whilst keeping all other covariates constant, reduced the risk of breastfeeding cessation by 13% (HR = 0.87, 95% CI = 0.85, 0.89). Only maternal age, as a confounder barely reached significance, indicating older mothers to be less likely to breastfeed for longer time periods. A mother being one year older increased the risk of breastfeeding cessation by 5% (HR = 1.05, 95% CI = 1.01, 1.09). No other covariates were significant (see Table 2). Sensitivity analysis without the covariate maternal age did not change pattern of results (see Table S6 [Online_Supplement.pdf]).

**Table 2:** Probability of breastfeeding cessation

Predictors	Coefficient	HR (95% CI)
IIFAS-G score	-0.13^***^	0.87 (0.85,0.89)
age child	< -0.01	1.00 (1.00,1.00)
age mother	0.05^*^	1.05 (1.01,1.09)
gestational age	0.04	1.04 (0.99,1.08)
education	-0.29	0.75 (0.51,1.10)
working	0.22	1.25 (0.83,1.90)

Mothers (*N* = 318) with a higher IIFAS-G have a reduced risk of breastfeeding cessation, whereas mothers, that are older have an increased risk of breastfeeding cessation

^*^p < 0.05; ^**^p < 0.01; ^***^p < 0.001

**Fig. 2:**
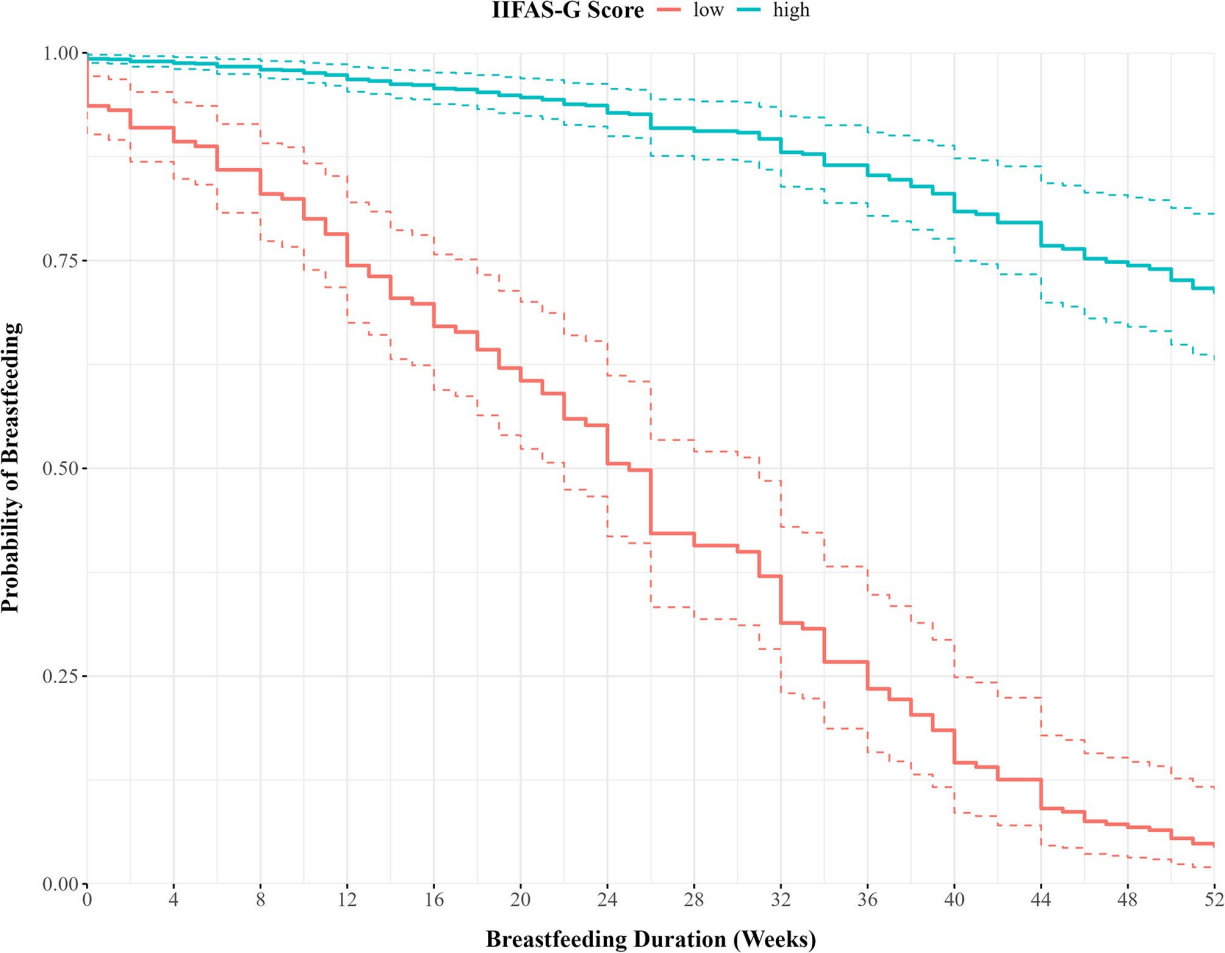
Probability of breastfeeding cessation given IIFAS-G. Probability of breastfeeding in the first year of life. Mothers who had exceptionally positive attitudes towards breastfeeding (1 SD above average), were significantly more likely to continue breastfeeding up to one year of life, when compared with mother who had very low attitudes towards breastfeeding (1 SD below average)

The probability of breastfeeding for mothers with high scores in the IIFAS-G ( ≥ + 1SD) at 6 months was between 90 and 97.5%, whereas for mothers with low values ( ≤ − 1SD) the probability to breastfeed was between 45 and 65%. This difference becomes even larger at 12 months of age. At that time, around two thirds (0.70–0.80) of mothers with positive attitudes are giving human milk to their child, in contrast the probability for mothers with more negative attitudes sink to less than 10% (0.03–0.10). Figure 2 shows the probability of breastfeeding over time for mothers being one standard deviation above and below the mean breastfeeding attitudes.

Likewise, a multiple logistic regression revealed IIFAS-G (*β* = 0.51, *p* < 0.001) and child age (*β* = 0.31, *p* < 0.001) to be associated with exclusive breastfeeding duration (see Table 3). Sensitivity analysis without the covariate maternal age did not change pattern of results see Table S5 [Online_Supplement.pdf].

**Table 3:** Effects of IIFAS-G score and maternal socio-demographic variables on exclusive breastfeeding duration assessed with multiple logistic regression

	b	SE	β	t
(Intercept)	-11.36	13.87		-0.82
IIFAS-G score	0.97	0.11	0.51	8.55^***^
age child	0.03	0.01	0.31	4.43^***^
age mother	-0.19	0.22	-0.05	-0.88
gestational age	-0.45	0.29	-0.09	-1.55
education	-3.66	2.06	0.11	-1.78
working	0.25	2.33	0.01	0.11

*N* = 213. ^***^p < 0.001

## Discussion

This study reports on the psychometric properties of the newly translated German version of the IIFAS. In this study, we validated the newly translated German version of the IIFAS, the IIFAS-G, in a sample of German-speaking mothers, analysing both factor structure and criterion validity. The use of the present validated version allowed us to demonstrate that positive attitudes towards breastfeeding in the collective studied is associated with almost 100% adherence to the 6-month breastfeeding recommendations of the WHO.

### Factor structure

To analyse whether the German version of the IIFAS assesses the same construct as the original version [32] or other translations of the IIFAS [35, 38–41], a CFA was fitted assuming the two latent factors “favourable towards breastfeeding” and “favourable towards formula-feeding”. Although the original model with 17 items displayed inadequate fit, a reduced 13-item version of the IIFAS demonstrated satisfactory model fit and high reliability. This reduced version is in accordance with the Hungarian, Japanese, Arabic and Persian translations of the IIFAS, as well as the English version administered in Singapore and Canada. All these administrations of the IIFAS scale have excluded at least one of the items also removed in our version [30, 35, 38–40, 42]. Ungváry et al. [35] analysed several shortened versions of the IIFAS with high internal consistency. Items that were consistently kept among different translations were identified as measuring social environment, factual beliefs (e.g., Item 4 “Breast milk is lacking in iron”) or generally accepted statements (e.g., Item 12 “Breastmilk is the ideal food for babies”). The items removed in our version failed to meet these criteria. Moreover, considering that the IIFAS was originally developed over two decades ago in the US, it is likely that since then many countries have undergone a number of institutional (e.g., Baby Friendly Hospital Initiative (BFHI)) and social (e.g. breastfeeding mothers at work) changes that may have influenced knowledge and attitudes towards breastfeeding [43]. The removed Item 8 “Women should not breastfeed in public places such as restaurants” and Item 17 “A mother who occasionally drinks alcohol should not breastfeed” are embedded within social norms and cultural environment [44–46]. These items usually evoke answers with little variance and poor fit across many different cultures and are thus oftentimes removed in other translations [30, 35, 36, 38, 42]. Item 16 “Breast milk is less expensive than formula” leads to almost universal acceptance, as it states financial facts rather than attitudes [35, 39]. In addition, Item 11 “Fathers feel left out if a mother breastfeeds” was poorly associated to maternal attitudes toward breastfeeding, which is consistent with a previous study [39]. We assume that Item 11, along with the other items removed, are less associated with the rest of the scale. The other statements are more related to nutritional values of human milk (e.g., Item 4 “Breast milk is lacking in iron.”) or convenience (e.g., Item 6 “Formula-feeding is the better choice if the mother plans to work outside the home”), and thus the removed items may contribute less to a homogeneous construct. Moreover, we assume that Item 11 may be more closely related to social norms or support, which alongside attitudes towards breastfeeding influence breastfeeding behavior and duration [47].

In our sample items 2 “Breastfeeding is more convenient than formula” and 15 “formula feeding is more convenient than breastfeeding” show high error term covariance, what we interpreted in being worded very similarly. Other administrations have also found a connection of those two items, even identifying them as a possible third factor “convenience” in exploratory factor analyses [40, 41].

### Criterion validity

To validate our IIFAS-G we used the “known-group comparison” to measure criterion validity. In our sample, mothers that were exclusively breastfeeding their child showed significantly higher IIFAS-G scores than mothers that were (additionally) formula-feeding. This effect was also present for mothers that have already stopped breastfeeding their child. The IIFAS-G was also associated with a longer duration of exclusive breastfeeding. These results are in line with other studies consistently showing that higher IIFAS scores predict mode of feeding and breastfeeding duration. [27, 30, 41]. The IIFAS is usually administered prenatally or right after birth to predict future breastfeeding behaviour. Our results suggest that, in addition, the IIFAS-G may also be administered retrospectively, after the child no longer receives human milk.

Furthermore, positive attitudes towards breastfeeding in our sample was associated with almost 100% adherence to the 6-month breastfeeding recommendations of the WHO. These findings are especially relevant, when considering that initiation rates of breastfeeding in German speaking countries are very high [5, 6]. This emphasizes the importance of attitudes as a key factor in supporting mothers and families to prolong breastfeeding periods, especially given that these effects are independent of other known factors affecting breastfeeding duration (e.g., maternal age). So far, there have only been few studies investigating how attitudes may influence long-term breastfeeding behavior [19, 22]. However, a cohort in rural Australia has shown that higher attitudes towards breastfeeding, measured with the IIFAS predicted exclusive breastfeeding at 6 months and prolonged breastfeeding at 12 months [27]. In order to meet the WHO recommendations for breastfeeding duration, future interventions focusing on modifiable psychological variables, such as attitudes, are in need of validated instruments to measure changes reliably. We propose the IIFAS-G as a validated tool to measure effects of attitudes towards breastfeeding in German-speaking populations.

### Limitations

This study proposes that the IIFAS-G is a reliable and valid instrument to measure attitudes towards breastfeeding. However, its cross-sectional design does not allow measuring the actual predictive qualities of the IIFAS-G score, which needs to be demonstrated in further studies. In particular, studies should control for the medical histories of both infant and mother, which could have potentially influenced their decisions to initiate breastfeeding (e.g., due to prematurity or medication) or to stop breastfeeding (e.g., post-partum depression), while simultaneously affecting attitudes towards breastfeeding. Furthermore, we administered the scale postpartum, potentially introducing bias due to prior experiences influencing attitudes toward breastfeeding which potentially could have influenced the factor structure of the scale. Future studies, thus, need to test for measurement invariance of the IIFAS-G in parents with and without own prior breastfeeding experiences.

Also in our sample we included mothers of children up to 1.5 years of life. Whilst it includes mothers who are breastfeeding for longer than average, it may also induce a potential recall bias for mothers that have stopped breastfeeding early and potentially answering the questionnaire more than a year after breastfeeding cessation. However, the risk of a recall bias will remain in all studies that are cross-sectional and include mothers that are currently breastfeeding, have stopped breastfeeding or have never breastfed. To allocate for this risk, a longitudinal approach could illuminate potential change in attitudes over a longer time period after breastfeeding cessation.

In addition, our results are drawn from a sample of highly educated and financially well-off mothers, although the high percentage of mothers with a migration background demonstrates some diversity. This raises the issue of a representation bias [48]. Since many studies have found effects of socio-demographic variables (e.g., socio-economic status) on breastfeeding practices [8, 21, 49], it is likely that attitudes towards breastfeeding differ in mothers with lower socio-economic status (e.g., due to lower education and available support). Especially, higher maternal education was consistently associated with higher IIFAS scores in other administrations of the IIFAS in different cultural contexts [27, 36, 38, 40, 42]. It is possible that criterion validity may be affected by this representation bias, since socio-economic status is also related to breastfeeding duration [11, 14, 49]. Thus, to ensure generalizability, future research needs to assess criterion validity in more diverse and representative samples, including tests of measurement invariance across different levels of socio-economic status. Furthermore, additional future studies should apply different measures to assess criterion validity and construct validity by assessing convergent and divergent associations with familiar constructs. For instance, higher attitudes towards breastfeeding should be related to initial higher intention to breastfeed and higher breastfeeding self-efficacy [50–52].

Lastly, it is noteworthy that the spoken German language differs between Switzerland, Germany and Austria, with many variations in grammar and dialects. It is possible that there are slight differences in the perception and understanding of the items between the three countries. To control for this, a larger sample with more participants from Germany and Austria is needed. However, since the written language remains the same in all three countries, we assume a bias of interpretation due to differences in spoken language to be minimal.

## Conclusion

The IIFAS-G is a reliable and validated instrument to assess maternal attitudes towards breastfeeding and formula-feeding in German-speaking countries.

### Electronic supplementary material

Below is the link to the electronic supplementary material.

Supplementary Material 1

## Data Availability

The dataset used and analysed during the current study are available from the corresponding author on reasonable request.
