# Supplementary material for: Reliability and validity of the German version of the Iowa infant feeding attitude scale (IIFAS-G) and relations to breastfeeding duration and feeding method
Source: Int Breastfeed J. 2024 Aug 21;19:58. doi: 10.1186/s13006-024-00665-6 (PMC11337571; doi:10.1186/s13006-024-00665-6)
Supplement: Supplementary file 1 — Supplementary Material 1 [file 13006_2024_665_MOESM1_ESM.pdf]

Online Supplement

This section includes supplementary information and analyses to the study "Reliability and Validity of the German Version of the Iowa Infant Feeding Attitude Scale (IIFAS-G) and relations to breastfeeding duration and feeding method".

Supplement A contains questionnaires used in the study, as well as mean and standard deviation of IIFAS-G. Supplement B contains additional unsatisfactory model fit, Cronbach's α of IIFAS-G and sensitivity analysis for criterion validity.

Supplement A

Table S1 Current or past feeding method

Are you (or your partner) currently breastfeeding your child or did you (or your partner) ever breastfeed your child?	1= child is still breastfed<br>2= child is weaned<br>3= child was never breastfed
Are you feeding your child with formula? Or have you ever fed formula to your child?	1= yes<br>2= no
Are you exclusively breastfeeding? Meaning you do not feed formula additionally?	1= yes<br>2= no
How are you giving milk to your child?	1= exclusively breastfeeding<br>2= mostly breastfeeding, some pumped milk with the bottle<br>3= approximately half breastfeeding, half pumped milk with the bottle<br>4= mostly pumped milk with the bottle<br>5= mostly pumped milk with the bottle
For how long was your child breastfed? Breastfeeding includes pumping milk and giving it with the bottle.	_____ months<br>_____weeks
Within that period how long was your child exclusively breastfed? Meaning no additional feeding with formula?	_____months<br>_____weeks

Table S2 Mean and standard deviation of translated IIFAS-G

Item	4	M	SD
1[a]	O[b]*: The nutritional benefits of breast milk last only until the baby is weaned from breast milk.*	3.6	1.10
	G[b]: Der ernährungsbedingte Vorteil von Muttermilch hält nur so lange an, bis das Baby von der Muttermilch entwöhnt ist.		
2[a]	O: *Formula-feeding is more convenient than breastfeeding.*	3.8	1.17
	G. Muttermilchersatz/Formula ist praktischer als zu stillen.		
3	O: *Breastfeeding increases mother-infant bonding.*	4.3	.98
	G: Stillen erhöht die Bindung zwischen der Mutter und dem Säugling.		
4[a]	O: *Breast milk is lacking in iron.*	3.8	.98
	G: Muttermilch fehlt es an Eisen.		
5	O: *Formula-fed babies are more likely to be overfed than are breast-fed babies.*	2.9	.97
	G: Babys, die mit Muttermilchersatz/Formula gefüttert werden, werden eher überfüttert, als Babys, die gestillt werden.		
6[a]	O: *Formula-feeding is the better choice if a mother plans to work outside the home.*	3.3	1.08
	G: Muttermilchersatz/Formula ist die bessere Wahl, wenn eine Mutter plant außerhalb der Wohnung zu arbeiten.		
7	O: *Breastfeeding is one of the joys of motherhood.*	2.7	1.20
	G: Mütter, die Muttermilchersatz/Formula füttern, verpassen eine der größten Freuden von Mutterschaft.		
8[a,c]	O: *Women should not breastfeed in public places such as restaurants.*	4.7	.68
	G: An öffentlichen Orten wie Restaurants sollten Mütter nicht stillen.		
9	O: *Babies fed breast milk are healthier than babies who are fed formula.*	3.2	1.19
	G: Babys, die gestillt werden, sind gesünder als Babys, die mit Muttermilchersatz/Formula gefüttert werden.		
10[a]	O: *Breast-fed babies are more likely to be overfed than formula-fed babies.*	4.0	.91
	G: Babys, die gestillt werden, werden eher überfüttert als Babys, die mit Muttermilchersatz/Formula gefüttert.		
11[a,c]	O: *Fathers feel left out if a mother breastfeeds.*	3.3	1.04
	G: Väter fühlen sich ausgeschlossen, wenn die Mutter stillt.		

12	O: *Breast milk is the ideal food for babies.*	4.6	.67
	G: Muttermilch ist die ideale Nahrung für Babys.		
13	O: *Breast milk is more easily digested than formula.*	3.7	.99
	G: Muttermilch ist leichter verdaulich als Muttermilchersatz/Formula.		
14[a]	O: *Formula is as healthy for an infant as breast milk.*	3.2	.98
	G: Muttermilchersatz/Formula ist für einen Säugling genauso gesund wie Muttermilch.		
15	O: *Breastfeeding is more convenient than formula feeding.*	4.0	1.03
	G: Stillen ist praktischer als Muttermilchersatz/Formula.		
16[c]	O: *Breast milk is less expensive than formula.*	4.6	.70
	G: Muttermilch ist billiger als Muttermilchersatz/Formula.		
17[a,c]	O: *A mother who occasionally drinks alcohol should not breastfeed her baby.*	2.5	1.24
	G: Eine Mutter, die gelegentlich Alkohol trinkt, sollte ihr Baby nicht stillen		

[a] Reverse-coded item.
[b] O: Original IIFAS (1).G: Translated IIFAS-G
[c] Item excluded from final version, due to low factor loadings.

Supplement B

Table S3 Unsatisfactory Model Fit of translated 17-item version

	Loading	SE	z-value	P(>|z|)
**Favorable towards breastfeeding**				
Item 3	.65	.06	11.27	.000
Item 5	.54	.05	10.29	.000
Item 7	.80	.05	14.96	.000
Item 9	.83	.06	14.28	.000
Item 12	.47	.05	10.53	.000
Item 13	.47	.05	9.02	.000
Item 15	.74	.06	12.42	.000
Item 16	.18	.05	3.47	.001
**Favorable towards formula-feeding**				
Item 1	.49	.07	7.46	.000
Item 2	.97	.05	18.72	.000
Item 4	.34	.05	6.30	.000
Item 6	.65	.06	10.63	.000
Item 8	.19	.05	3.71	.000
Item 10	.38	.06	6.78	.000
Item 11	.18	.07	2.66	.008
Item 14	.54	.06	9.50	.000
Item 17	.11	.07	1.55	.122

Model with two latent factors, 17 items. CFI= 0.83; TLI = 0.81; RMSEA = 0.08.

Table S4 Item-total correlation and Cronbach's alpha if item is dropped for 13-item solution

	Internal Reliability	
**Item**	**corrected item-total correlation**	**Cronbach's α (if item deleted)**
1[a]	.48	.82
2[a]	.75	.80
3	.61	.81
4[a]	.41	.82
5	.57	.81
6[a]	.61	.81
7	.63	.81
9	.68	.80
10[a]	.46	.82
12	.66	.81
13	.48	.82
14[a]	.61	.81
15	.69	.80

[a] = reverse coded item

Table S5 Sensitivity analysis for multiple logistic regression: Effects of IIFAS-G score and socio-demographic variables on exclusive breastfeeding duration

	**B**	**SE**	***β***	***t***
(Intercept)	-14.89	11.67		-1.28
IIFAS-G score	.92	.11	.48	8.29***
age child	.03	.01	.27	3.95***
gestational age	-.44	.29	-.09	-1.55
education	-3.03	1.86	-.09	-1.63
working	.14	2.25	< .00	.06

Due to high number of missing values on maternal age (n = 30) we conducted a sensitivity analysis to ensure effect pattern remain the same.

*** $p < .001$

$N = 234$

Table S6 Sensitivity analysis for cox-regression: probability of breastfeeding cessation

Predictors	Coefficient	HR (95% CI)
IIFAS-G score	-.12***	.88 (.88–.90)
age child	<-.00	1.00 (1.00–1.00)
gestational age	.03	1.03 (.99–1.07)
education	-.21	.81 (.58–1.13)
working	.38	1.46 (.97–2.19)

Due to high number of missing values on maternal age (n = 30) we conducted a sensitivity analysis to ensure effect pattern remain the same.
* $p < 0.05$ **; $^{*}p < .01$ ***; $p < .001$
N = 347

References

1. De la Mora A, Russell, D. W., Dungy, C. I., Losch, M., & Dusdieker, L. The Iowa Infant Feeding Attitude Scale: analysis of reliability and validity. Journal of Applied Social Psychology. 1999;29(11):2362-80.
